# Seroprevalence of 12 serovars of pathogenic *Leptospira* in red foxes (*Vulpes vulpes*) in Poland

**DOI:** 10.1186/s13028-018-0388-2

**Published:** 2018-05-31

**Authors:** Jacek Żmudzki, Zbigniew Arent, Artur Jabłoński, Agnieszka Nowak, Sylwia Zębek, Agnieszka Stolarek, Łukasz Bocian, Adam Brzana, Zygmunt Pejsak

**Affiliations:** 1 Swine Diseases Department, National Veterinary Research Institute, Partyzantow 57, 24-100 Pulawy, Poland; 2 University Centre of Veterinary Medicine UJ-UR, University of Agriculture in Krakow, Mickiewicza 24/28, 30-059 Krakow, Poland; 3 Epidemiology and Risk Assessment Department, National Veterinary Research Institute, Partyzantow 57, 24-100 Pulawy, Poland; 4 Veterinary Hygiene Research Station, Wroclawska 170, 45-836 Opole, Poland

**Keywords:** Leptospirosis, Prevalence, Red fox, Serology, *Vulpes vulpes*, Zoonosis

## Abstract

**Background:**

*Leptospira* spp. infect humans and a wide range of domestic and wild animals, but certain species such as small rodents and red foxes (*Vulpes vulpes*) play a particular role as reservoirs and transmission of leptospirosis as they easily adapt to many habitats including human environments. To investigate the significance of red foxes in the epidemiology of leptospirosis in Poland, a seroprevalence survey was conducted. During the 2014–2015 hunting season, blood samples of 2134 red foxes originating from the central-eastern part of Poland were collected. Serum samples were tested by a microscopic agglutination test for the presence of specific antibodies to *Leptospira* serovars Icterohaemorrhagiae, Grippotyphosa, Sejroe, Tarassovi, Pomona, Canicola, Hardjo, Ballum, Australis, Bataviae, Saxkoebing and Poi.

**Results:**

Antibodies to at least one serovar were detected in 561 sera (26.3%). The highest seroprevalence was found in the Subcarpathia (41.6%) and Warmia-Masuria (40.3%) provinces. Antibodies were mainly directed against serovars Poi (12.4%), Saxkoebing (11.3%), and Sejroe (6.0%).

**Conclusions:**

Exposure of red foxes to certain *Leptospira* serovars seems to be common in central and eastern Poland. In addition, the high prevalence of antibodies against *Leptospira* spp. in foxes may indicate a potential risk of infection for humans and other species coming into contact with these animals.

## Background

Leptospirosis caused by pathogenic spirochetes of the genus *Leptospira* is an important but sometimes neglected infection that affects people and animals worldwide. Leptospirosis is a re-emerging major public health problem in many countries and is one of the most widespread zoonoses. It is an excellent example validating the “One Health” approach, where the relationship between humans, animals and ecosystems needs to be considered in order to better understand and manage a disease [1]. Some serovars of *Leptospira* can chronically infect domestic and wild animals and in particular small rodents. In addition to rodents, other wild animal species such as the red fox (*Vulpes vulpes*) may act as a reservoir [2]. The bacteria are occasionally transmitted through direct contact with mammal hosts, but the majority are usually transmitted via contact with contaminated soil and water [3], where leptospires’ survival outside the host is favoured by warm moist conditions [4]. The red fox lives throughout Europe, mainly inhabiting forests, meadows, coastal dunes and urbanized areas [5]. The Polish hunting statistics for 2015 indicate that the population of red foxes in Poland is 190,000–200,000 individuals, with a tendency to remain stable [6]. Red foxes prey upon small rodents, among other animals and the red fox may transmit leptospirosis to humans. A recent study indicate that small mammals might be an important source of human leptospirosis as both rodents and humans share infections caused by *Leptospira* spp. from the same serogroups [7]. The aim of the present study was to determine the seroprevalence for *Leptospira* spp. in red foxes from central and eastern Poland.

## Methods

### Sample collection and study area

**Fig. 1:**
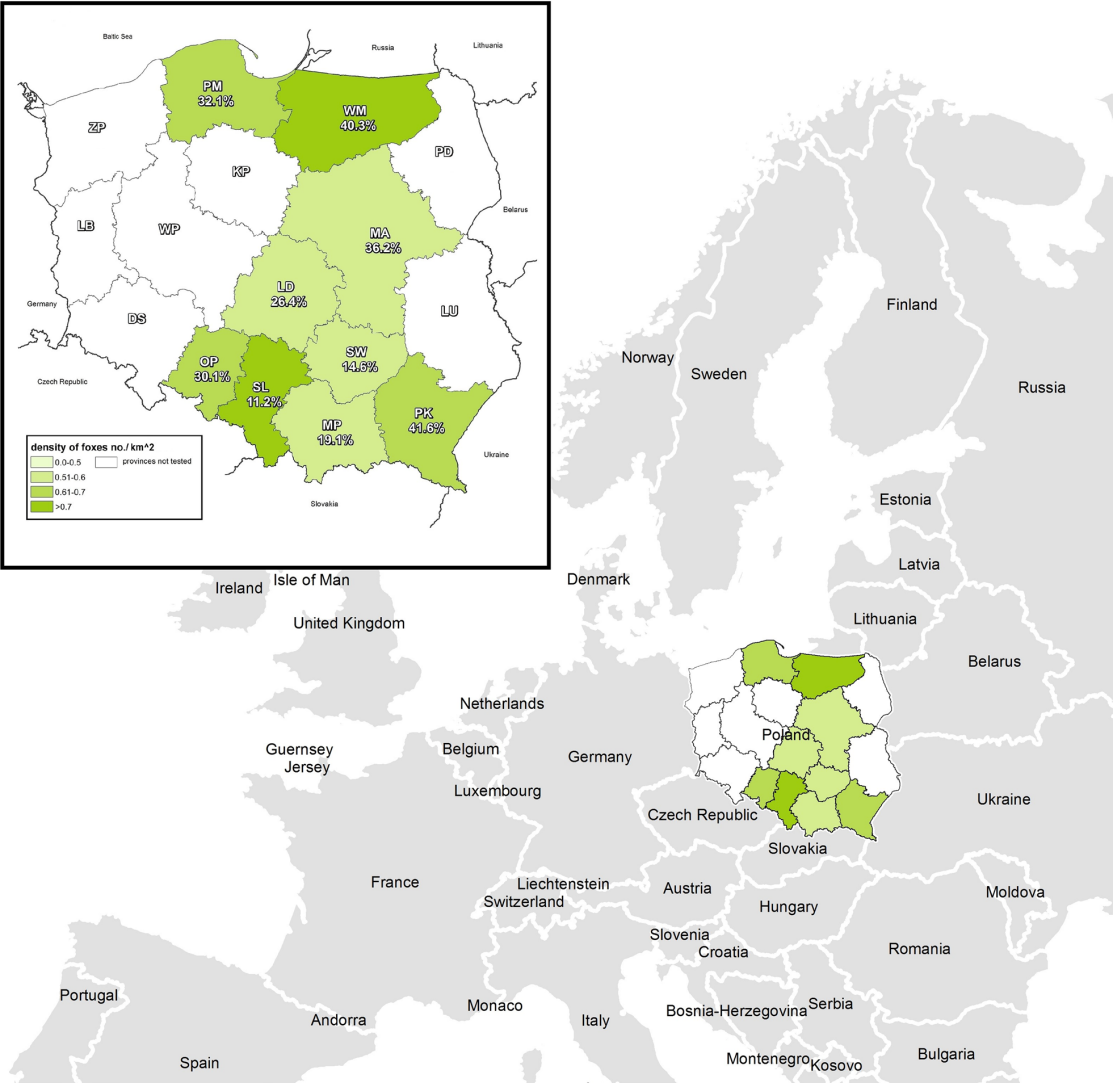
Geographic distribution of red foxes seropositive for pathogenic *Leptospira* in Poland. *LD* Łódzkie, *MP* Lesser Poland, *MA* Masovia, *OP* Opolskie, *PK* Subcarpathia, *PM* Pomerania, *SL* Silesia, *SW* Świętokrzyskie, *WM* Warmia-Masuria, *DS* Lower Silesia, *KP* Kuyavian-Pomerania, *LB* Lubuskie, *LU* Lubelskie, *PD* Podlaskie, *WP* Greater Poland, *ZP* West Pomerania

Blood samples from red foxes (n = 2134) were collected during the 2014–2015 hunting seasons in Poland. Blood was taken from the thoracic cavity or heart of animals culled primarily through the rabies monitoring program. Sex and geographic location were recorded and age was determined by the degree of dentine surface wear and tooth eruption (juveniles: < 1 year; mature > 1 year) (Table 1). The samples originated from 134 counties of nine provinces of Poland and were mainly collected from the central and eastern (49–55°N, 17–23°E) parts of the country (Fig. 1). Blood samples were centrifuged at 4500 *g* for 30 min and serum stored at − 20 °C until analysis.

**Table 1:** Total number of red foxes from Poland hunted in 9 Polish provinces between 2014 and 2015

Sex	Females						Males						Unknown	Total	No of seropositive	% of anti-*Leptospira* sp. antibodies positive (95% CI)
Age	Adult		Young	Unknown			Adult		Young		Unknown		Unknown			
Result (*Leptospira* sp.)	−	+	−	+	−	+	−	+	−	+	−	+	−			
Province/season																
LD	45	14	35	14			81	27	26	12				254	67	26.4 (21.1–32.3)
Spring	12	4	3				11	4	4	1				39	9	
Autumn	12	2	9	1			15	3	7					49	6	
Winter	21	8	23	13			55	20	15	11				166	52	
MP	56	18	60	8			92	23	51	12				320	61	19.1 (14.9–23.8)
Unknown	36	10	37	3			75	13	28	5				207	31	
Spring	17	6	5	2			12	6	7	2				57	16	
Autumn	3	2	18	3			5	4	16	5				56	14	
MA					47	30					80	42		199	72	36.2 (29.5–43.3)
Unknown					47	30					80	42		199	72	
OP	25	13	29	6			42	24	25	9				173	52	30.1 (23.3–37.5)
Summer	4	1	11	3			7	5	11	3				45	12	
Autumn	21	12	18	3			35	19	14	6				128	40	
PK			19	17		1			38	24	2			101	42	41.6 (31.9–51.8)
Unknown			19	17		1			38	24	2			101	42	
PM	43	17	7	4			36	16	3	5				131	42	32.1 (24.2–40.8)
Winter	43	17	7	4			36	16	3	5				131	42	
SL	61	11	62	8			106	8	55	9			1	321	36	11.2 (8.0–15.2)
Spring	7	2	3				9	2	5	1			1	30	5	
Summer	13	2	22	4			18	3	17	5				84	14	
Autumn	23	5	28	1			48	2	22	2				131	10	
Winter	18	2	9	3			31	1	11	1				76	7	
SW	40	8	73	11			73	14	36	5				260	38	14.6 (10.6–19.5)
Spring	8	1	5				5	2						21	3	
Summer	5	2	13				10	3	9					42	5	
Autumn	3	1	27	7			15	3	10	3				69	14	
Winter	24	4	28	4			43	6	17	2				128	16	
WM	73	36	30	26			87	70	34	19				375	151	40.3 (35.3–45.4)
Winter	73	36	30	26			87	70	34	19				375	151	
Total	343	117	315	94	47	31	517	182	268	95	82	42	1	2134	561	26.3 (24.4–28.2)

*LD* Łódzkie, *MP* Leser Poland, *MA* Masovia, *OP* Opolskie, *PK* Subcarpathia, *PM* Pomerania, *SL* Silesia, *SW* Świętokrzyskie, *WM* Warmia-Masuria

### Microscopic agglutination test

Serum samples were tested by a microscopic agglutination test (MAT) using a range of 12 *Leptospira* serovars representative of 10 serogroups found in Europe: Icterohaemorrhagiae (RGA strain, representing the Icterohaemorrhagiae serogroup), Grippotyphosa (Moskva V strain, Grippotyphosa serogroup), Sejroe (M84 strain, Sejroe serogroup), Tarassovi (Perepelicyn strain, Tarassovi serogroup), Pomona (Pomona strain, Pomona serogroup), Canicola (Hond Utrecht IV strain, Canicola serogroup), Hardjo (Hardjoprajitno strain, Sejroe serogroup), Ballum (MUS127 strain, Ballum serogroup), Australis (Ballico strain, Australis serogroup), Bataviae (Swart strain, Bataviae serogroup), Saxkoebing (MUS 24 strain, Sejroe serogroup) and Poi (Poi strain, Javanica serogroup) [8, 9]. The selection of the serovars used was based on their common identification in previous European studies [10–13] reporting *Leptospira* spp. in wild carnivores.

Each serovar was grown in 10 mL of Ellinghausen–McCullough–Johnson–Harris (EMJH) medium, at 30 ± 1 °C for at least 4 but no more than 8 days depending on the serovar. The concentration of bacteria was adjusted to 1–2 × 10^8^ cells/mL using a Helber counting chamber. The sera were initially diluted 1:50 and screened for antibodies to the 12 serovars. A volume of each antigen equal to the diluted serum volume was added to each well with a final serum dilution of 1:100 in the screening test. The final concentration of antigen after mixing with the diluted serum was 1–2 × 10^4^ cells/mL. The plates were incubated at 30 ± 1 °C for 2–4 h and subsequently examined by dark-field microscopy. The titre was defined as the highest dilution where ≥ 50% of the antigen suspension added to the tested serum was agglutinated. When agglutination was observed, the relevant sera were end-point tested using twofold dilutions ranging from 1:100 to 1:25,600.

The quality control of the MAT was performed by using certified reference *Leptospira* strains and anti-*Leptospira* rabbit antisera (Veterinary Sciences Division, AFBI, OIE Leptospira Reference Laboratories, Belfast, and the WHO/FAO and National Collaborating Centre for Reference and Research on Leptospirosis, Royal Tropical Institute (KIT), Amsterdam, the Netherlands). Testing of the samples was conducted at the National Reference Laboratory of Leptospirosis, National Veterinary Research Institute in Pulawy, Poland using an accredited method according to PN/EN ISO/IEC 17025-2005.

### Statistical analysis

Statistical analysis was used to study the impact of the season, sex, age, region and population density of foxes on *Leptospira* seroprevalence. It was based on logistic regression models to describe the influence of several variables *X*_1_, *X*_2_, …, *X*_*n*_ on the dichotomous variable *Y*:

$$P\left( {Y = 1\left| {x_{1} } \right.,x_{2} , \ldots ,x_{n} } \right) = \frac{{e^{{\left( {\beta_{0} + \sum\nolimits_{i = 1}^{n} {\beta_{i} } x_{i} } \right)}} }}{{1 + e^{{\left( {\beta_{0} + \sum\nolimits_{i = 1}^{n} {\beta_{i} } x_{i} } \right)}} }}$$

where β_*i*_ is the regression coefficient for *i* = 0, …, *n*, *χ*_*i*_ are independent variables (measurable or qualitative) for *i* = 1, 2, …, *n*.

The maximum likelihood method was used to estimate the model’s coefficients. The Wald test was used to evaluate the significance of individual variables. Evaluation of model fit to data was performed using the likelihood ratio (LR) test.

Five predictors (4 qualitative and 1 quantitative) were included in the modelling:

- *sampling season* (spring: March–May, summer: June–August, autumn: September–November, or winter: December–Feburary);
- *sex* (male, female);
- *age* (young, adult);
- *province* (LD: Łódzkie; MP: Lesser Poland; MA: Masovia; OP: Opolskie; PK: Subcarpathia; PM: Pomerania; SL: Silesia; SW: Świętokrzyskie; WM: Warmia-Masuria); (Fig. 1) and
- *fox density in counties in 2015* (No/km^2^).

The dependent variable was the qualitative result of the study. Analysis was performed for results without distinguishing between serovars (*Leptospira* spp.: positive/negative) and for each serovar separately. The selection of variables for modelling was based on analytical stepping methods (step-wise). For qualitative variables, 0–1 coding for k − 1 variables was used (Table 2).

**Table 2:** Dichotomous coding for qualitative variables with an example of sampling season

Sampling season	Spring	Autumn	Winter
Spring	1	0	0
Summer	0	0	0
Autumn	0	1	0
Winter	0	0	1

The following classes of variables were reference classes in models: ‘summer’ for *sampling season*, ‘female’ for *sex*, ‘young’ for *age* and ‘SL’ for province. Parameters of significant and best fit logistic regression models obtained for each analysis are shown in Table 3. The accepted significance level was alpha = 0.05. STATISTICA data analysis software in version 10 (StatSoft, Inc.) and ArcGIS 10.4.1 for Desktop Standard (ESRI, Inc.) were used for statistical and spatial data analysis. Red fox demographics were derived from the Polish Hunting Association-PZL [6].

**Table 3:** Results of the best fit logistic regression models obtained for each analysis

Significance assessment of model (*P* value of LR test)	Independent variable	Coefficient (*β*_*i*_)	Std. error	P value (Wald)	Odds ratio	Confidence OR − 95%	Confidence OR + 95%
Models for infection of *Leptospira* sp. (without distinction of serovars)							
< 0.001	Absolute term (*β*_0_)	− 2.92912	0.296482	< 0.001	0.05	0.03	0.10
	LD	1.216036	0.233494	< 0.001	3.37	2.13	5.33
	MP	0.671037	0.228562	0.003	1.96	1.25	3.06
	MA	1.68051	0.237135	< 0.001	5.37	3.37	8.55
	OP	1.388372	0.247953	< 0.001	4.01	2.46	6.52
	PK	1.769046	0.269941	< 0.001	5.87	3.45	9.96
	PM	1.534127	0.265823	< 0.001	4.64	2.75	7.81
	SW	0.555254	0.259964	0.03	1.74	1.05	2.90
	WM	1.630786	0.20659	< 0.001	5.11	3.41	7.66
	Fox density (No/km^2^)	1.142803	0.307487	< 0.001	3.14	1.72	5.73
< 0.001	Absolute term (*β*_0_)	− 1.50766	0.198255	< 0.001	0.22	0.15	0.33
	Spring	0.267965	0.280004	0.34	1.31	0.75	2.26
	Autumn	0.0834	0.232275	0.72	1.09	0.69	1.71
	Winter	0.688467	0.211402	0.001	1.99	1.31	3.01
Model for Icterohaemorrhagiae							
0.003	Absolute term (*β*_0_)	− 6.41457	0.839692	< 0.001	0.002	0.0003	0.008
	Fox density (No/km^2^)	2.913659	0.989553	0.003	18.42	2.65	128.30
	Adult	− 1.18961	0.553268	0.03	0.30	0.10	0.90
Model for Grippotyphosa							
0.001	Absolute term (*β*_0_)	− 5.71115	0.543301	< 0.001	0.003	0.001	0.01
	Fox density (No/km^2^)	2.364823	0.677533	< 0.001	10.64	2.82	40.19
Model for Sejroe							
0.015	Absolute term (*β*_0_)	− 3.43721	0.318798	< 0.001	0.03	0.02	0.06
	LD	1.130284	0.386552	0.003	3.10	1.45	6.61
	MP	0.35268	0.419714	0.40	1.42	0.62	3.24
	MA	0.85591	0.422493	0.04	2.35	1.03	5.39
	OP	0.110974	0.514159	0.83	1.12	0.41	3.06
	PK	1.228934	0.461089	0.008	3.42	1.38	8.44
	PM	1.047612	0.44818	0.02	2.85	1.18	6.87
	SW	0.408686	0.434724	0.35	1.50	0.64	3.53
	WM	0.880843	0.376223	0.02	2.41	1.15	5.05
Model for Australis							
< 0.001	Absolute term (*β*_0_)	− 6.36907	0.610643	< 0.001	0.002	0.0005	0.01
	Fox density (No/km^2^)	2.843724	0.730836	< 0.001	17.18	4.10	72.02
Models for Saxkoebing							
0.024	Absolute term (*β*_0_)	− 2.77882	0.325736	< 0.001	0.06	0.03	0.12
	Spring	0.445929	0.436438	0.31	1.56	0.66	3.68
	Autumn	0.408323	0.368228	0.27	1.50	0.73	3.10
	Winter	0.806557	0.34165	0.02	2.24	1.15	4.38
< 0.001	Absolute term (*β*_0_)	− 2.94772	0.25661	< 0.001	0.05	0.03	0.09
	LD	1.010782	0.318737	0.002	2.75	1.47	5.13
	MP	0.715284	0.318663	0.03	2.04	1.09	3.81
	MA	1.257746	0.322546	< 0.001	3.52	1.87	6.62
	OP	1.021486	0.343397	0.003	2.78	1.42	5.45
	PK	1.939495	0.341159	< 0.001	6.96	3.56	13.58
	PM	1.452948	0.341859	< 0.001	4.28	2.19	8.36
	SW	− 0.63976	0.461137	0.17	0.53	0.21	1.30
	WM	1.121314	0.296979	< 0.001	3.07	1.71	5.49
Models for Poi							
< 0.001	Absolute term (*β*_0_)	− 5.45234	0.689411	< 0.001	0.004	0.001	0.02
	LD	2.814176	0.617742	< 0.001	16.68	4.97	56.01
	MP	1.032509	0.682165	0.13	2.81	0.74	10.70
	MA	3.445862	0.612244	< 0.001	31.37	9.44	104.22
	OP	3.293047	0.618209	< 0.001	26.92	8.01	90.51
	PK	2.239957	0.687842	0.001	9.39	2.44	36.19
	PM	2.960175	0.643965	< 0.001	19.30	5.46	68.24
	SW	2.610502	0.629299	< 0.001	13.61	3.96	46.74
	WM	3.666819	0.591781	< 0.001	39.13	12.26	124.88
	Fox density (No/km^2^)	1.043369	0.476866	0.03	2.84	1.11	7.23
< 0.001	Absolute term (*β*_0_)	− 2.89037	0.342638	< 0.001	0.06	0.03	0.11
	Spring	0.375612	0.464447	0.42	1.46	0.59	3.62
	Autumn	0.519876	0.383324	0.18	1.68	0.79	3.57
	Winter	1.368756	0.353764	< 0.001	3.93	1.96	7.87
0.003	Absolute term (*β*_0_)	− 2.30544	0.125369	0	0.10	0.08	0.13
	Adult	0.437116	0.152208	0.004	1.55	1.15	2.09

*LD* Łódzkie, *MP* Leser Poland, *MA* Masovia, *OP* Opolskie, *PK* Subcarpathia, *PM* Pomerania, *SW* Świętokrzyskie, *WM* Warmia-Masuria

## Results

Antibodies against a *Leptospira* serovar was found in 561 serum samples (26.3%). The highest seroprevalence was observed in foxes hunted in the Subcarpathia (41.6%) and Warmia-Masuria provinces (40.3%) (Table 1, Fig. 1). Specific antibodies were mainly directed against Poi (12.4%), Saxkoebing (11.3%), and Sejroe (6.0%) serovars with serum antibody titres up to 1:25,600 in individual animals (Table 4). When analysing the logistic regression model of positive and negative serostatus (excluding data related to individual *Leptospira* serovars), a significant influence of the area (province) and associated density of foxes on the serostatus was found. The model showed that all provinces had significantly greater odds for having seropositive foxes than the reference SL province, in which the lowest percentage of seropositive foxes was observed. The highest odds ratio (OR = 5.87) with the highest seroprevalence was shown for the PK province. In addition, with an increase of fox density by one animal per km^2^, the probability of detecting seropositive animals increased more than threefold and it almost doubled in winter when compared to summer. However due to data deficiencies e.g. sampling date, seasonal influence on the obtained serological results was analysed using a separate logistic regression model.

**Table 4:** Distribution of pathogenic *Leptospira* antibody titers for 561 positive red foxes hunted during season 2014–2015 in Poland

Serovar	No of antibody-positive samples (%)										Prevalence of serovar (95% CI) (%)
	1:100	1:200	1:400	1:800	1:1600	1:3200	1:6400	1:12800	1:25600	Total	
Icterohaemorrhagiae	8 (0.4)	3 (0.1)	3 (0.1)	1 (0.05)	2 (0.1)	1 (0.05)	0	0	0	18	0.8 (0.5–1.3)
Grippotyphosa	6 (0.3)	16 (0.75)	8 (0.4)	4 (0.2)	1 (0.05)	2 (0.1)	0	0	0	37	1.7 (1.2–2.4)
Sejroe	39 (1.8)	37 (1.7)	30 (1.4)	16 (0.75)	2 (0.1)	2 (0.1)	0	0	1 (0.05)	127	6.0 (5.0–7.0)
Tarassovi	0	1 (0.05)	0	0	0	0	0	0	0	1	0.1 (0.0–0.3)
Pomona	7 (1.5)	7 (1.5)	8 (0.4)	8 (0.4)	2 (0.1)	2 (0.1)	0	0	0	34	1.6 (1.1–2.2)
Canicola	0	2 (0.1)	1 (0.05)	0	0	0	0	0	0	3	0.1 (0.0–0.4)
Australis	7 (1.5)	11 (0.5)	1 (0.05)	7 (1.5)	2 (0.1)	0	0	0	0	28	1.3 (0.9–1.9)
Saxkoebing	63 (3.0)	55 (2.6)	66 (3.1)	44 (2.1)	8 (0.4)	3 (0.1)	2 (0.1)	0	1 (0.05)	242	11.3 (10.0–12.8)
Ballum	0	1 (0.05)	1 (0.05)	1 (0.05)	0	0	0	0	0	3	0.1 (0.0–0.4)
Poi	64 (3.0)	68 (3.2)	63 (3.0)	34 (1.6)	11 (0.5)	19 (0.9)	4 (0.2)	1 (0.05)	1 (0.05)	265	12.4 (11.1–13.9)
Bataviae	1 (0.05)	1 (0.05)	3 (0.1)	3 (0.1)	0	1 (0.05)	0	0	0	9	0.4 (0.2–0.8)
Hardjo	0	2 (0.1)	0	1 (0.05)	0	0	1 (0.05)	0	0	4	0.2 (0.1–0.5)

Based on analyses for individual serovars, an increase of fox density by one animal per km^2^ increased the risk of being seropositive by 2.8, 10.6, 17.2 and 18.4 times for the serovars Poi, Grippotyphosa, Australis and Icterohaemorrhagiae, respectively. The models also show a significant influence of the province on the proportion of seropositive samples. A significantly higher risk of being seropositive to Sejroe serovar was observed in the LD (OR = 3.1), MA (OR = 2.4), PK (OR = 3.4), PM (OR = 2.9) and WM (OR = 2.4) provinces compared to the SL province.

When compared to the reference SL province, antibodies to the Saxkoebing and Poi serovars were more prevalent in foxes from all provinces except SW (OR from 2.0 to 7.0), and MP province (OR from 9.4 to 39.1) respectively. An impact of the season on the seroprevalence to particular serovars was observed. Antibodies against serovars Saxkoebing and Poi were ~ 2 and 4 times more frequent, respectively, during the winter period than during summer. The age of the foxes influenced the serostatus for some serovars such as Icterohaemorrhagiae that was detected more frequently in young foxes (OR = 3.3) and Poi found more often in adults (OR = 1.5) (Table 3). Using a one-factor model the association between influence of sex on serostatus was not significant (LR-test P = 0.0525, OR = 1.44, 95% CI 0.99–2.09).

## Discussion

Other serological surveys have shown that red foxes are frequently exposed to *Leptospira* spp. of different serovars [10, 11, 13]. However this is the first prevalence study on the occurrence of antibodies to a broad range of *Leptospira* serovars in a red fox population in eastern Europe. The high seroprevalence (26.3%) in red foxes in Poland is comparable to that found in Spain (47.1%) [10] and Croatia (31.3%) [13] but higher than in other European countries such as Germany (1.9%) [14] and Norway (9.9%) [11]. Hypothetically any pathogenic *Leptospira* may infect domestic and wild animals, but in practice only a small number of serovars are endemic in any particular region.

Antibodies against serovar Poi were the most commonly detected. Exposure of foxes to this serovar is not surprising given the results of previous Polish studies where serogroup Javanica (to which serovar Poi belongs) was also reported in horses, goats, and sheep [15–17]. Besides serovar Poi, antibodies against serovar Sejroe were also prevalent in foxes. This is consistent with other studies as serovars Hardjo, Sejroe and Saxkoebing (all belonging to the Sejroe serogroup) are widely prevalent in animals in Europe [18–21]. MAT reactions to serovar Hardjo commonly detected in sheep and cattle [18–20, 22, 23] were not common in foxes. The presence of seropositive animals to this serogroup could be mainly attributed to Sejroe or Saxkoebing serovars (Table 4). It may be associated with fox diet as the main source of food for red foxes are wild small mammals, which are known reservoirs of Saxkoebing and Sejroe serovars [24]. Antibodies to Sejroe serogroup were previously detected in pigs, dogs, horses and cattle in Poland confirming a widespread exposure of different animal species to leptospires from this serogroup [15, 25–28]. In addition, this indicates an endemic occurrence of this serovar and a possible role of the environment in pathogen transmission. The observed regional differences in exposure to different *Leptospira* serovars may be related to active circulation of *Leptospira* spp. in the environment [12].

Studies conducted in other European countries provide scientific evidences that the most common serovar among red foxes is serovar Icterohaemorrhagiae [10, 11, 13], which however seems to be rare in the Polish red fox population (Table 4). As leptospires are sensitive to desiccation, the regional differences in climate conditions may have a significant influence on seroprevalence in general or for some serovars in particular. In that aspect, Poland differs from other countries such as Spain and Croatia where the seroprevalence of *Leptospira* spp. in foxes has been investigated [10, 13].

Although the studies were conducted on a reasonable number of hunted animals originating from different locations across the country, the number of tested serum samples of red foxes did not fully reflect the size of the animal population present in the studied provinces. It could be taken as a major limitation to interpretation of the occurrence and prevalence of tested *Leptospira* serovars in the Polish population of red foxes. Nevertheless, the findings still provide useful data on the seroepidemiology of red foxes exposed to different *Leptospira* serovars in this part of Europe and their role as an important source of zoonotic *Leptospira* spp. for humans.

## Conclusions

Red foxes of central and eastern Poland, particularly in the Subcarpathia and Warmia-Masuria regions, are highly exposed to *Leptospira* spp. Due to the high prevalence of foxes, their predatory behaviour and their varied diet mainly composed of small mammals, they could be considered as sentinel animals of environmental contamination with leptospires. Interactions between animals require further epidemiological investigations to elucidate the role of wild carnivores as a reservoir of rarely occurring *Leptospira* serovars pathogenic for other animals and humans.

## Abbreviations

DS: Lower Silesia
EMJH: Ellinghausen–McCullough–Johnson–Harris medium
KP: Kuyavian-Pomerania
LB: Lubuskie
LD: Łódzkie
LR: likelihood ratio
LU: Lubelskie
MA: Masovia
MAT: microscopic agglutination test
MP: Lesser Poland
OP: Opolskie
OR: odds ratio
PD: Podlaskie
PK: Subcarpathia
PM: Pomerania
SL: Silesia
SW: Świętokrzyskie
WM: Warmia-Masuria
WP: Greater Poland
ZP: West Pomerania
